# Phenomenological analysis of going home in Caribbean-American international travelers

**DOI:** 10.1186/s40794-015-0016-3

**Published:** 2015-12-20

**Authors:** Koya C. Allen

**Affiliations:** 1 Department of Biostatistics, Environmental Health & Epidemiology, Kent State University College of Public Health, PO Box 5190, Kent, OH 44242 USA; 2 Present Address: Counter Bio-threats Cell, Force Health Protection, J42 Medical Readiness Division, US European Command Headquarters, US Department of Defense, Stuttgart, Germany

**Keywords:** Cultural embeddedness, Mosquito avoidance practices, Visiting friends and relatives, International travel behavior, Dengue prevention

## Abstract

**Background:**

In travel health risk assessments visiting friends and relatives (VFR) travel status is often used as an indicator for high-risk travel behavior. VFR travelers have been associated with increased risk of travel-associated illnesses due to poor adherence to travel guidelines and lack of pre-travel health consultations. For travelers to dengue endemic regions, guidelines include compliance with mosquito avoidance practices (MAP). The goal of this study is to understand the meaning of travel experiences to the home country for immigrant and first generation American VFR travelers in the United States (US).

**Methods:**

A phenomenology study was conducted on VFR travelers to identify social and physical environmental factors associated with travel health behaviors, and determine how ‘going home’ influences compliance with recommendations for dengue prevention. Purposive sampling identified participants for semi-structured interviews on travel behavior with iterative collection and analysis until data reached saturation.

**Results:**

Interviews revealed five themes that defined the experience of going home: connectedness; control of the experience; two different experiences at home; seeing what home has to offer; and there is no place like home. Moreover, risk perception of health and disease risks in the travel destination influenced travel behavior and compliance with guidelines.

**Conclusions:**

VFR travel status does not fully capture the experience of international travel. Behavior was associated with the emergent concept of *Cultural Embeddedness* when traveling home and to new destinations. More research on improving terminology for travel health risk assessments is needed to improve prevention strategies in VFR travelers.

## Background

Individual behavior using mosquito avoidance practices (MAP) is the primary strategy offered to international travelers for prevention of mosquito-borne diseases such as dengue fever (DF). As one of the most common arboviral diseases worldwide, understanding influential factors of DF acquisition in susceptible populations is essential in control and management of the disease. Allen (2013) determined that compliance with MAP guidelines was a result of group influences and social interaction across different international travel experiences [1]. This association of behavior with the social environment can be described by the important concept of adaptation, used in Piagetian theory. Piagetian theory discusses the role of interchanges between an organism and the environment; occurs on a constant basis, and contributes to the process of construction for cognitive development [2]. Investigation of the role of culture on use of MAP during travel, can utilize this theoretical concept to increase understanding of interpersonal interactions, as an aspect of the social environment during travel, which may hold significant influences on actual MAP. Moreover, phenomenological research methods can reveal why and how compliance with travel health recommendations during travel to an individual’s home country may be different from travel to a novel or foreign destination.

Dengue in the Americas has reemerged as a major health concern [3, 4]. Dengue is one of the most common travel-associated infections in returning travelers and is the second most common cause of febrile illness in travelers after malaria [5, 6]. In addition, dengue is a significantly underreported disease associated with travel due to limited surveillance and nonspecific, self-limiting symptomatology in primary infections in adult travelers [5–7]. The primary prevention strategy for dengue in travelers is MAP. This includes: use of insect repellent, spatial repellents or insecticides with an US Environmental Protection Agency (EPA)- registered active ingredient such as DEET, Picaridin, Oil of Eucalyptus or IR3535; wearing protective clothing (light-colored, long sleeves and long pants); awareness of mosquito-biting behavior; and killing visible mosquitoes [8].

An important risk factor for acquisition of mosquito-borne diseases during travel is lack of adherence to guidelines for use of MAP [9, 10]. Additionally, poor health-seeking behavior exists amongst travelers; particularly those visiting friends and relatives (VFR) travelers, repeat travelers and immigrant travelers or US children of foreign-born parents returning to their country of origin [11]. Furthermore, risk perception for traveling to the home country or country of origin may inhibit objective thinking for health risks since home is sometimes synonymous with going to a safe and familiar place. VFR travelers tend to have low-risk perception or misperceptions of prevention methods and have been significantly associated with poor compliance to travel health recommendations in the past [11–13].

Investigation of compliance behaviors for MAP is necessary for advancements in pre-travel health interventions and safety during travel [14]. However, before any interventions for improved compliance behaviors can be developed, it is necessary to understand influences and challenges to compliance behaviors in high-risk travel populations. A high-risk region for mosquito-borne diseases is the Caribbean. Countries within the Caribbean region have high endemicity rates of dengue. In addition, there is a high frequency of travel between Caribbean countries and the US. Increases in travel, and imported dengue from the region present an important travel health risk for Caribbean VFR travelers and the possibility for secondary transmission in the continental US.

### The problem with VFR

In travel medicine, the classification of VFR travelers has been widely used in different contexts. Studies have used the term VFR as a categorical approach to understanding risk of disease acquisition among all travelers planning to visit friends and relatives during international travel (e.g., [11, 12, 15–17]). The VFR terminology inadvertently assumed that the traveler was an immigrant traveling home to a developing country. The redefined VFR framework improved applicability to other travelers by eliminating ethnicity, and solely used intent to visit friends and relatives with a corresponding risk assessment of determinants of health [18]. As a result, anyone who self-identifies travel purpose as VFR to an international destination is classified as a VFR traveler.

There are conflicting ideas regarding the elimination of ethnicity or nationality from risk assessments. For some, a lack of adequate information regarding potential risk may exist because ethnicity, as a proxy for culture, may provide further insight into the broader meanings specific to the ‘cultural lens’ that travelers view health. Additionally, VFR travel status indicates an activity, and does not incorporate the importance of the cultural lens each individual traveler uses when going abroad. Furthermore, culture may influence compliance to recommendations for specific travel health behaviors that may be associated with disease risk.

Alternatively, for dengue prevention in travelers, MAP may differ for each individual based on external influences with less significant similarities specific to their membership within a particular cultural group. This concept of ethnicity in dengue prevention behaviors exemplifies the correlation between an individual’s culture, their travel experiences, and the cultural lens through which they view that experience. The relationship between ethnicity and culture can also influence the social interactions that may be significantly associated to behavior outcomes and therefore disease risk. Despite ethnic or cultural classifications, such as Caribbean-American, and VFR, travelers each have individual lived experiences when going home or to their country of origin that are specific to them. Relevant themes that emerge within this context are simply similarities that may exist across people within a culture. Actual behavior combines the inherent learned behaviors relevant to culture and ethnicity, with cues to action from social or physical environmental influences on preventative health behaviors.

From this viewpoint, ethnicity should not be a category that implies a set of behaviors; rather, acknowledging the culture of these travelers would help to understand their worldview and the influences that culture has on their behaviors. Moreover, while ethnicity or nationality does not solely define culture, it can be an important aspect of a risk assessment because it may offer some insight to the culture of that individual. Furthermore, in order to understand actual MAP as a travel health preventative health behavior, it is necessary to understand how the traveler’s culture and the meaning of ‘going home’ influences the overall travel experience that will take place and influence MAP.

## Methods

A phenomenological investigative approach was used to understand the meaning of the lived experience of traveling home. An existential-phenomenological model focuses this study in the understanding and describing of the human experience [19]. The thematic structure of a lived experience then describes the phenomena in question. Rather than studying behavior that results from experimental situations, past experiences- in ‘specific ecologically significant situations’- reflect the participant’s worldview through a naturalistic paradigm [20].

### Data collection and analysis

In order to meet the aim of describing the lived experience of the VFR traveler, and following phenomenological research methods, this study identified a sample population that could be considered high-risk based on regional dengue statistics and past travel health research on VFR travelers. The sample population characteristics were U.S. travelers to the Caribbean who are of Caribbean decent, including Caribbean-Americans who emigrated during childhood, and first-generation Caribbean-Americans. This population is considered unique and appropriate because VFR research has shown similar concerns for immigrants returning home and first generation Americans.

The data format used was semi-structured qualitative interviews [21, 22]. Inquiry on the experience of going home and going to other destinations allowed participants to describe both types of travel so that the analyses could delineate the differences, if or when present, between going home and visiting another international destination. Purposive sampling was used to identify and recruit participants who met the criteria for this study. Inclusion criteria for research participants were: (1) Adult currently living in the U.S.; (2) Caribbean-American (of Caribbean or West-Indian descent including islands of the Caribbean and Lesser Antilles); and, (3) traveled to their home country or country of heritage**.** The sampling frame for participants included those individuals who had participated in previous studies that identified factors of influence on international travel behavior within this population [1]. While a limiting factor, focusing the purposive sampling strategy through previous research grounded the context of the current study in a wider population where knowledge, attitudes and practices of MAP were already assessed [23].

An iterative data collection and analysis process was implemented; data saturation occurred after five interviews and interviewing stopped after the eighth interview. Each audio recording was reviewed and transcribed prior to analysis. The data collection and analysis process was iterative in accordance with the three stages of interpretive phenomenology; fore-understanding, interrogation, and reflection. Data analysis incorporated the latter two stages, and served to compare budding themes across and within groups to present commonalities and shared concepts [24]. Five interviews were audio-recorded with the participants’ consent. Two interviews took place by phone and one interview took place over social media. The latter three interviews were not audio-recorded since they were not conducted in-person. For all interviews, post-interview field notes were written to record initial thoughts and details about the interview. During the phone interviews, in depth field notes were taken, in addition to post-interview field notes. Interviews followed a semi-structured format using an interview guide as needed, but allowed for narrative responses from participants using probe questions to elicit additional details. Interviews ranged from approximately 30 min to 1 h.

Hycner [24] guidelines were used as guidance for analysis of interview transcripts. After transcription of each interview, the transcripts were read and memos recorded of presuppositions, with an effort, but not expectation of ‘*pure objectivity*.’ Units or codes were delineated for a general meaning from the verbatim statements of the participant, followed by appropriating meaning relevant to the research question in the context of the phenomenon under investigation. Where relevant, paralinguistic cues and common use of phrases or words for significance to that individual’s meanings were noted. Clustering of statements were used to present relevant meanings and contributed to overarching themes. A summarizing view of the themes of the interview revealed the experience of that individual. If additional questions or an incomplete view seemed apparent, the participant was contacted to ask additional questions. Final contextualization of the subthemes from all interviews was used to explain the broader themes and noted varieties across participants [24].

Recruitment, participation and analyses were conducted in accordance with the Kent State University Institutional Review Board (IRB) policies for research on human subjects’ through protocol #12-334. This included informed consent by participants and anonymity of participants in their corresponding interviews.

## Results

### Main theme of international travel

**Fig. 1:**
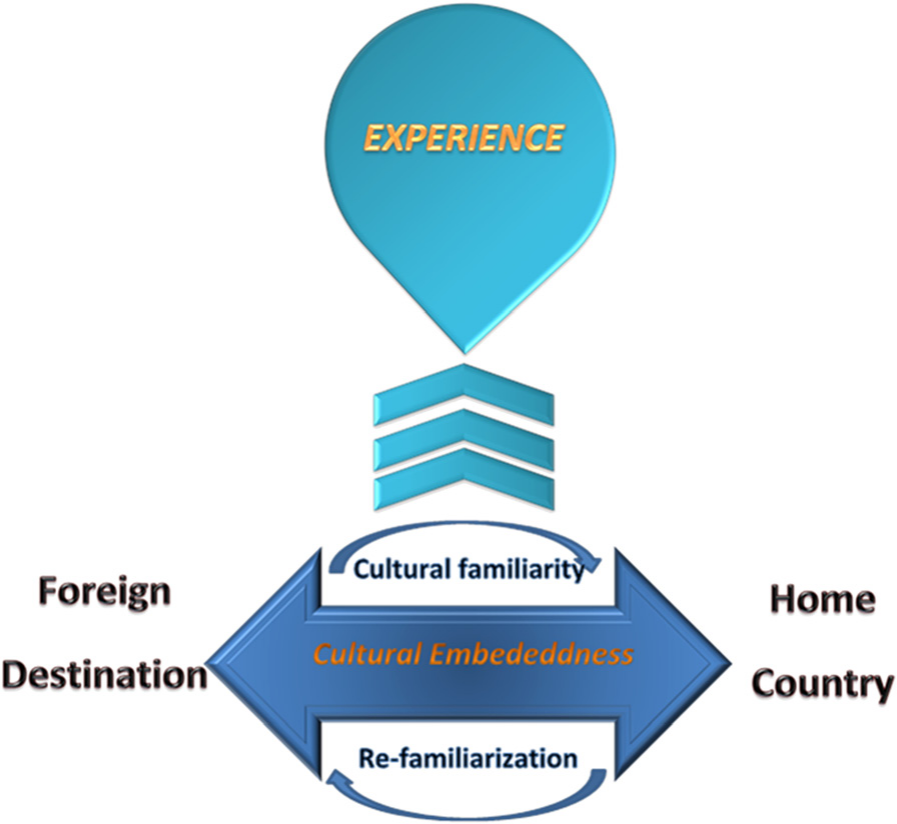
The Experience of International Travel. International travel entails the process of Cultural Embeddedness where re-familiarization and cultural familiarity acquaint the traveler with the new foreign destination or the home country through the travel experience

The analysis of interview data revealed five themes that fit under the broader concept of the travel ‘*experience’* sought by travelers. Whether traveling to the home country or traveling to another international travel destination, the focus was on the ‘*experience;*’ the excitement and goal to experience what the travel destination has to offer. This overarching theme differed between the excitement of experiencing the unknown in a new or foreign travel destination and the excitement of experiencing the known or familiarities of the home country. This goal, to experience a place, created a cyclical process of understanding for travelers through re-familiarization and cultural familiarity. When going to the home country, travelers achieved re-familiarization through exploration of their home/country of descent with the approach of experiencing it as a foreign destination. When traveling to a new travel destination, travelers often had the goal to experience the foreign country as a local in an effort to become familiar and learn about the culture of the travel destination. In the ‘*culturally embedded’* traveler, this experience could lead to feeling at home in a new travel destination (Fig. 1).

### Themes of going home

Themes that define the ‘*experience’* of international travel for going home are: Connectedness; Control of the Travel Experience: Childhood versus Adulthood; Two Different Experiences at Home; Seeing What Home has to Offer; and, There is No Place Like Home. Each theme and subtheme is described below; all names have been changed to maintain the anonymity of participants. In total, there were 8 participants; age range 25–54, 50 % US born, 75 % with heritage in Republic of Trinidad and Tobago, 75 % female. The slightly skewed proportion of female travelers and travelers with heritage in Republic of Trinidad & Tobago was a result of the sampling method utilizing individuals who participated in the previous study on KSA’s for dengue in the Caribbean (see [1, 23]). Each theme is described below using some quotes as an example of the participant responses and the extrapolation of meaning from their experiences.

#### Connectedness

Connectedness to the home country was an important aspect of the overall travel experience. Each of the travelers discussed the importance of their connection to the country of origin/country of descent through pride and appreciation. Their country was described as an aspect of who they are and how they understand who they are. Regardless of where they live or travel, a piece of home and their culture is always a part of them. The connection to the home country also set the stage for the experiences traveling elsewhere. Two participants, who we will refer to as Naomi and Stella, discussed the connection to home as making the travel experience to other international destinations different. They stated that they are more connected to their home country because it is their heritage. Stella articulated the difference in travel experience simply with the statement that travel elsewhere “*is not like going home (Stella).”* Another participant, Ava, described the connectedness as a part of the broader experience of life. She said that traveling to the country of her heritage makes her *“feel connected to something more”* and that it helps to *“understand where you come from”* because it explains who you are.

#### Subtheme: sharing the connection

A subtheme of connectedness included the desire to share the feeling and experience of going home with your loved ones. Sharing the experience with children born in the U.S., spouses or friends was an important aspect of sharing a piece of you with them. In the case of children, sharing meant helping them to understand a piece of themselves through exposure to their country of heritage. This may emerge as the same connectedness to the home country when they are adults.

#### Subtheme: seeing my home country

Seeing the home country was also a major component of experiencing the home country. Particularly for the first generation Caribbean-American travelers, an important aspect of the experience was the need to see the connection. One participant, Zoey, described her first time visiting Trinidad as “*a great experience”* because she could see the island of her heritage. Seeing the home country, figuratively, was important as well. It allowed participants to witness their culture, which helped them to understand themselves better. Another participant, Brody, described this aspect of seeing in his description of visiting Trinidad. He said, “*Growing up in a Caribbean home and community [in the U.S.] is watered down…when I go to Trini[dad] it’s pure in its finest form (Brody).*”

#### Control of the travel experience: childhood versus adulthood

The experience traveling to the home country was different for travel as a child versus travel as an adult. The travelers expressed this difference as the ability to have control over the experience. The experience as a child meant lack of control over the travel activities, time of year for travel, and ability to make decisions for the overall travel experience. Emily discussed the difference in experience as a child versus adult in her behavior. When talking about eating street food and taking precautions for health, Emily said, “*I don’t buy as much on the street though…now that I’m an adult I understand that it’s not very safe (Emily).”* Other participants described the difference in travel as a child versus an adult in the ability to understand and “*appreciate the experience more (Peyton).”*

#### Subtheme: don’t need a reason

A part of traveling to the home country as an adult included the realization that they do not need a reason to travel home. Rather than traveling for a specific event or during a certain time of year, travel could be anytime for any reason. This was the farthest extent to which the traveler could exert control over the travel experience as an adult, by no longer needing a reason to travel home.

#### Two different experiences

An extension of the previous theme ‘control over the experience,’ is the distinction between ways to experience the home country. The travelers all indicated that there are different travel purposes when visiting the home country. All of these travelers can be VFR travelers; however, the travel purpose of visiting friends and relatives was sometimes an underlying component to the travel experience and not always the main travel purpose. Trinidadian participants all made the distinction between travels to the home country for Carnival versus traveling to the home country for other reasons. The experience traveling to Carnival was different from traveling for vacation, to VFR, or during another holiday such as Christmas or Borough Day. One participant, Lucas, described the difference between activities, length of stay and purpose. He said that when he goes for Carnival it is a time for him to “*have fun, to make merry and just to enjoy the place in a short space of time*.” He compared this to his experience going to Trinidad for vacation, where the experience is “*calm*” and there is “*family time*.” The participants, Lucas and Emily, described the Carnival experience as “*different*” and “*intense*.” Naomi explained, “*It’s different during Carnival”* as compared to other times of year when she *“can actually visit relatives (Naomi).”*

The participants from other Caribbean countries also discussed the different experiences of going to their country of descent when the travel purpose was to a specific event as compared to a holiday. Many of the participants described the lack of time or ability to VFR during the Carnival experience. One participant, Lucas, summed up his thoughts visiting for Carnival versus visiting for vacation by saying *“it’s like two different experiences and feelings…but it’s always amazing (Lucas).”*

#### Subtheme: the Carnival getaway

The constant travel experience across all participants regardless of Caribbean country of origin was Trinidad Carnival. By design, all of the participants experienced Trinidad Carnival in recent years. The Carnival experience was different from the experience of going to any other travel destination and different for the travelers whose home country is Trinidad. Carnival was often described as a means for travelers to get away from the stress of everyday life. Brody explained his “*plan to go every year*” and get away by saying “*Carnival is a great stress reliever…its clean fun (Brody)*.” In addition to explaining how Carnival was “*very different than anything I was ever used to (Emily),*” Emily also explained how the Carnival experience was a getaway for her. She said she was looking for a vacation with friends *“because I was going through a lot personally at the time and I really just wanted to get away (Emily)*.” Despite the view that the Carnival experience is a getaway or stress reliever, the experience is also intense and full of activities. Many participants described the intensity and euphoria of the Carnival experience, as being *“like a drug,” “absolutely amazing,”* and “*the greatest time of your life.”*

#### What my country has to offer

The cycle of the goal to experience travel can begin here for travelers to the home country. The goal to experience the home country was usually to experience it as though it were a new travel destination with the added benefit of familiarity. The traveler comes to a point of realization that the home country not only has a lot to offer, but that they need to experience their home country in order to strengthen their relationship and pride in the country. This often materialized as having a tourist experience in the home country. The tourist experience meant learning about the country, sightseeing and experiencing the culture as though you were visiting for the first time. The experience was an aspect of changing travel purpose between childhood and adulthood, and as a realization that the country could offer an exotic vacation in addition to friends and family. This concept could also be applied to the broader experience of travel to anywhere. Many of the participants viewed experiencing the culture and what a country “has to offer” as a part of their reason for travel to any travel destination. When Peyton travels, her goal is that she “*looks for being immersed in that city… [and] whatever they are known for (Peyton*).” What a country has to offer can include their landmarks, history, architecture and spirituality, as well as experiencing the culture of the place.

#### No place like home

The travelers all had expectations for a great experience when traveling to the home country. Each participant described their past travel experiences to the home country as being “*great*” or “*amazing.*” This facilitates the need and desire to return again in the future. Many of the participants already had set travel plans coming up, or plans that were in progress. When there were no plans in place, there was almost disbelief as to why not. Participants all had difficulty articulating the feeling of going to the home country as nothing less than great; often repeating that it is a great experience, feeling and being the best. This feeling and expectation is present regardless of the travel purpose. Sometimes the experience was great because of getting to VFR, for a celebration like Carnival, or simply because of the experience of being in the home country. The expectation of an amazing travel experience also created a preference to go home versus travel to other travel destinations when the traveler had to make a choice.

#### Subtheme: noticing the difference

Aside from the expectation of an amazing experience when going home, the change was sometimes unanticipated. The traveler may feel *“disconnected”* or like a *“foreigner”* at times because they have changed or are different because of their American cultural attributes. The differences observed included the lack of change in the home country; the traveler became aware of the difference between their home country and the US or the American lifestyle. Emily described her experience as inciting a feeling of familiarity, but also feeling *“a little disconnected.”* She said, *“everything looks different…I don’t know my way around anymore…so in a lot of ways I feel like a tourist (Emily)*.” Others described the lack of change by saying that “*everything was the same….there was no progress”* or explaining that the advances of American cities include access that leads to a different perception and way of life.

However, it seems when the travelers do visit their home country and experience both the familiarity and notice the differences, the experience enhances the excitement of going home again in the future, instills a new layer of familiarity through the new experiences, and renews pride in their home country.

#### Subtheme: i should just stay

Many of the participants thought about visiting more often or moving to the home country. After comparing the home country to America, acknowledging the differences, and rationalizing reasons for or against relocation, participants would devise a compromise to live in two places at once. The plan was for a retirement option, to maintain the dream of moving or at a minimum, visiting the home country more often. The deliberation included a description of the country feeling like home, planning to have a retirement home and traveling often or simply moving once it was feasible to do so. As one participant described the difficulty in leaving after each visit, the concluding thought and feeling was to stay in the home country permanently.

## Discussion

### Influence on travel health

Participants discussed their travel health behaviors within the spectrum of going home to going somewhere foreign. The experience revealed a range of comfort and familiarity with going home versus going somewhere new based on the presence of factors like family, travel companions, and cultural similarities.

The overarching concept of risk perception exemplified compliance with travel recommendations and overall travel behavior. All of the travelers, in varying degrees held the stance that “*it depends on where I’m going*” to decide whether any preventive health behaviors were necessary. Travelers with past travel experiences that included health issues held the most concern for pre-travel advice and preparation. The perception of risk was based on the previous experiences with healthcare or illness when abroad. Concerns for the level of healthcare outside of the US, and the need to be cautious in foreign destinations were only necessary depending on the travel destination.

The trend of awareness of health during travel preparations were among the participants who had health-related backgrounds. They described the different influences of their career on decisions surrounding health and travel. Another trend amongst participants was the receipt of pre-travel advice only when vaccinations were required for travel. Destinations that were considered foreign correlated with places that were both very far away and required immunizations and/or prophylaxis for travel; for example, many African countries.

Risk perception, depending on the travel destination, was important for MAP and dengue prevention. All of the participants knew of dengue, and were aware of how to decrease risk of acquiring dengue by use of MAP, particularly with insect repellent. There was some concern of dengue, but this concern was either short-lived or not enough to elicit long-term changes in behavior. When participants were aware of friends, family or reported deaths due to dengue they expressed concern, and some intent to change their use of MAP when traveling to the home country. However, others often had beliefs about their personal risk or risk in the home country for mosquito bites that would decrease the likelihood of behavior change to use MAP. Brody said, “*A good friend had a friend in Trini who passed away from that [Dengue] in January (Brody)*!” Hearing of the death, he was concerned about it for his upcoming travel to Trinidad because he “*didn’t know much about it*.” However, for taking extra precautions, he said “*no not really”*, he would *“just [use] some insect repellent*” because the mosquitoes “*love my sweet blood*.” Zoey described an experience getting sick while in Trinidad that may have been dengue. She said “*I was just like really weak… really tired…headache…but I’m not sure [if it was Dengue]*.” However, talking about a family member who had dengue and was “*really bad,*” she said, “*maybe I’ll try now…because I know I did not put on any type of bug repellent or anything…maybe I will do the bug repellent thing… so I don’t get the other 3 strains (Zoey).*”

Interestingly, despite awareness of dengue, there was also a perceived lack of control over prevention of dengue. Some were convinced that they would not be directly affected by dengue and some believed that they could become infected, but that they could not control the risk. For example, when asked about individual precautions for dengue, Ava said “*no not at all…it’s not an STD or chronic disease…It’s a mosquito…I don’t have much control over that…the mosquito is going to come bite me whether I do anything or not…it’s like…it’s life*.” Whereas Ava introduced the idea of perceived lack of control over dengue risk, in other cases, the participants discussed the different activities that would elicit MAP and the level of comfort that may exist and lead to low perception of risk for mosquito bites. The lack of a health risk perception in the home country was not a denial that there would be no health concerns; rather, without personal experiences, the need to prevent dengue or other health issues did not seem a critical aspect of travel planning.

The irony is that the overall experience in a foreign country was sometimes to experience the place like a local (e.g., visit homes, local food, and street food), yet the concept when going home for vacation was sometimes to see it as a tourist and do the type of activities that tourists do. What does this mean for health risks? How can we measure VFR and ‘going home’ as high-risk travel if those same individuals are doing low-risk activities at home and high-risk activities in foreign countries? However, better attempts at compliance to MAP seem to occur when individuals are traveling to someplace perceived as foreign and high-risk for disease. The problem for dengue is that even though everyone had some level of dengue knowledge, many were not concerned with the risk enough to take special precautions. Interestingly, despite logic that special precautions at home or in places where the traveler felt comfortable, were unnecessary, the travelers were usually aware that mosquitoes were present and they were at risk of being bitten.

### VFR status & Cultural Embeddedness

Culture is defined by Hoebel, as ‘an integrated system of learned behavior patterns which are characteristic of the members of a society, and which are not a result of biological inheritance [25].’ Drawing on the concepts of Hoebel’s definition, culture plays a significant role not from the standpoint of expected behaviors based on cultural classifications; rather, as a function of the social environment. Actual travel behavior using MAP therefore should not be the same as intended behaviors prior to travel; actual behavior should evolve based on some of the social interactions taking place during the actual travel experience. In addition, the intent of that individual should be a reflection of their culture as a whole. The intent would consider the travel destination, and the meaning of travel type, as interpreted by that individual. This concept was evident in the lived experiences of traveling to the home country described by the travelers in this study. Despite the traveler’s expectations or anticipation for travel home, their experiences were in part a result of travel purpose, and social environment, including travel activities, such as meals in someone’s home, Carnival fetes (parties), or time with family. The inherent cultural component of the experience, however, remained unchanged. Within the excitement of travel to somewhere foreign versus travel to the home destination, the traveler’s connection to the home country was an aspect of their culture. The goal to experience the culture of another country was an effort for that traveler to further understand others and themselves through the experience.

Regardless of perceptions, and known risks, the travelers all love to travel. Many of the travelers expressed the need for trips to the home country, and in some ways, chose home over other travel destinations. The decision-making that contributes to planned travel experiences is an important aspect of the concept of Cultural Embeddedness [1]. Choosing the home country for future travels over other destinations influences the intended and actual behaviors that may occur when going home because of the familiarity, knowledge, and perceptions developed- and possibly altered- with each travel experience to the home country. In the concept of Cultural Embeddedness, as travelers became more familiar with the travel destination and discover similarities or differences between themselves, their travel companions and the travel destination, they also learn about the culture of the travel destination. This interaction potentially leads to assimilation with certain aspects of the culture. The process of Cultural Embeddedness may occur in stages, from discovery or realization of similarities and differences, to a sense of cultural familiarity, a level of comfort, and then eventually becoming embedded in the culture of the travel destination.

Cultural Embeddedness is a theoretical concept that, with additional research, can be used as the foundation for improving travel health risk assessments. The concept speaks to the concern that culture is currently excluded in pre-travel health risk assessments with the current VFR travel definition and scope. As stated in the introductory section of this paper, ethnicity does not define culture; however, it provides a proxy for the individual’s worldview, which coupled with the lived experience, can shape health risk behaviors. Moreover, the VFR definition and travel behaviors exhibited in this study exemplify the possibility of high-risk travelers being low risk in the home country and high-risk in foreign destinations based on risk perception.

An important theme for influence on MAP was that it depends on the travel destination for many people to decide on their perception of health risks and need comply with recommendations (e.g. MAP). Often this meant more relaxed preventive behaviors in the home country, which is consistent with past research in travel medicine on VFR travelers [11, 16, 18]. However, an important contrast is that the relaxed preventive measures were not specific to VFR travel experiences. For many travelers, relaxed preventive methods were a function of risk perception for the travel destination and how comfortable the traveler felt once they arrived. Even in travel experiences to a new travel destination, depending on the level of comfort and the travel companions, the resultant behavior was dependent not on VFR status, but on level of comfort and Cultural Embeddedness. It is recommended that the current VFR definition be re-assessed to determine if risk assessments can improve by measuring Cultural Embeddedness as well as identifying VFR status. Additionally, innovative strategies employing this concept in current technological advances such as apps and on social media may improve access to high-risk populations and promote pre-travel consultation. For example, improving access to information surrounding travel health risks and prevention strategies through these platforms may aid in virtual or self-assessments regarding risks. During clinical pre- and post- travel consultations, a full assessment utilizing the Cultural Embeddedness concept may improve estimation of high-risk behaviors, and compliance with guidance for preventative behaviors during travel. Where possible, a pre- and post- consultation can allow a clinician to assess where on the Cultural Embeddedness spectrum the traveler is and may be used to guide individualized interventions for minimizing risky behaviors; however, more research is needed to identify an appropriate protocol for assessing this concept in a traveler. Additionally, the concept of Cultural Embeddedness can be used to improve understanding of disease risk and the potential relationship to travel behavior in travelers at-risk of acquiring and importing diseases of concern [26].

### Strengths and limitations

Understanding the overall experience of international travel holds important implications for travel behavior that are specific to the individual, culture, and the society to which a traveler is visiting. Limitations in this study included the skewed sample population, which was a reflection of the sampling strategy employed. Additionally, the study is limited in generalizability to other travel populations. The study focused on a very specific and small population within US travelers; however, the study represents a basis for exploring the context and impact that culture may have on travel health behaviors. Moreover, the study begins to delve into the bias that can exist within studies when population characteristics are deduced into specific categories for defining risk. Future research should explore this phenomenon in other groups of travelers, and for other preventive health behaviors for travel-associated health risks. For example, unintentional injuries, sexually transmitted infections, food-borne and water-borne diseases are sample topics that could be explored. Each of these health risks may present differently for priorities and meanings regarding preventive health behaviors for going home and for going to new international travel destinations. Furthermore, additional research is needed to understand the role of Cultural Embeddedness in decision-making for preventative strategies and health behaviors during international travel.

## Conclusions

Going to the home country has a special meaning different from travel to other travel destinations. The travel experience to the home country also can have a variety of different travel purposes and activities associated with the experience. For this reason, classifying all travelers that may have friends or relatives and possibly a connection to the country as VFR, makes it difficult to estimate true risk based on when or why they are traveling to the home country. The use of VFR status in travel health risk assessments does not capture the meaning of going home or the concept of Cultural Embeddedness. Travel purpose needs to be captured differently, and additional research should explore new terminology or estimations of travel health risk.
